# Patterns of Change in Functional Connectivity and Motor Performance Are Different in Youth Recently Recovered from Concussion

**DOI:** 10.1089/neur.2024.0122

**Published:** 2025-01-22

**Authors:** Nishta R. Amin, Mary Beth Nebel, Hsuan-Wei Chen, Tyler A. Busch, Elizabeth D. Rosenthal, Stewart Mostofsky, Stacy J. Suskauer, Adrian Svingos

**Affiliations:** ^1^ Brain Injury Clinical Research Center, Kennedy Krieger Institute, Baltimore, Maryland, USA.; ^2^ Center for Neurodevelopmental and Imaging Research, Kennedy Krieger Institute, Baltimore, Maryland, USA.; ^3^ Department of Neurology, Johns Hopkins University School of Medicine, Baltimore, Maryland, USA.; ^4^ Department of Psychiatry and Behavioral Sciences, Johns Hopkins University School of Medicine, Baltimore, Maryland, USA.; ^5^ Department of Physical Medicine and Rehabilitation, Johns Hopkins University School of Medicine, Baltimore, Maryland, USA.; ^6^ Department of Pediatrics, Johns Hopkins University School of Medicine, Baltimore, Maryland, USA.

**Keywords:** concussion, functional connectivity, adolescence, clinical recovery, longitudinal design, motor deficits

## Abstract

Adolescents who have sustained a concussion or mild traumatic brain injury (mTBI) are prone to repeat injuries which may be related to subtle motor deficits persisting after clinical recovery. Cross-sectional research has found that these deficits are associated with altered functional connectivity among somatomotor, dorsal attention, and default mode networks. However, our understanding of how these brain–behavior relationships change over time after clinical recovery is limited. In this study, we examined categorical and dimensional trajectories of functional connectivity and subtle motor performance in youth clinically recovered from mTBI and never-injured controls (10–17 years). All participants completed task-based and resting-state functional magnetic resonance imaging scans and the Physical and Neurological Examination of Subtle Signs (PANESS) at initial and 3-month follow-up visits. We examined somatomotor-dorsal attention and somatomotor-default mode network connectivity and their association with PANESS performance. Compared with controls, a larger proportion of youth recovered from mTBI showed increases in somatomotor-dorsal attention functional connectivity over time; in contrast, there were no differences in somatomotor-default mode connectivity trajectories between youth recovered from mTBI and controls. Relative to controls, youth recovered from mTBI who showed greater increases in somatomotor-dorsal attention connectivity over time also completed motor tasks more slowly at the 3-month compared with the initial visit. Collectively, these findings suggest that longitudinal changes in somatomotor-dorsal attention functional connectivity may be associated with lingering motor learning deficits after clinical recovery from pediatric mTBI. Further research is necessary to understand how trajectories of functional connectivity and motor performance can inform individual-level outcomes, for instance, susceptibility to future injuries in both youth who are never injured and those clinically recovered from mTBI.

## Introduction

Concussion or mild traumatic brain injury (mTBI) in youth deserves special attention because there is a high rate of injury in this age group and the developing brain is particularly susceptible to its effects. Adolescent athletes who have already incurred one concussion are more likely to sustain additional injuries following medical clearance to return to play.^1,2^ One contribution to injury risk may be that the developmentally beneficial tendency for adolescents to engage in risky behaviors has been shown to be exacerbated to unhealthy levels after sustaining an mTBI.^3,4^

Another reason for increased injury risk may be related to emerging evidence suggesting a dissociation between complete neurobiological recovery and symptom resolution at medical clearance to return to play in youth post-mTBI. Most adolescents recover from acute behavioral symptoms and are medically cleared to return to play within 1–3 months postinjury.^5,6^ However, some adolescents who appear fully recovered from mTBI, and are permitted to return to play, still exhibit subtle motor deficits. These deficits have been observed in gait and postural tasks,^7,8^ as well as in motor performance during cognitive-motor dual-task conditions.^9,10^ Recent research has found that the Revised Physical and Neurological Examination of Subtle Signs (PANESS)^11^ is a valuable assessment for identifying lingering speed and accuracy motor deficits in youth clinically recovered from mTBI.^12,13^ Neuromuscular control and attention are essential for completing the PANESS, and impairments in these skills have been associated with a heightened risk for neuromuscular injury independent of concussion history.^14,15^ Yet, these subtle motor skills are often not considered in return-to-play evaluations that tend to focus on cognitive assessments, symptom resolution, and some gross motor indicators.^16,17^ Existing tools used to determine recovery may lack sufficient sensitivity to detect subtle motor signs post-mTBI.

Parallel to persistent subtle motor signs, there is also evidence of persistent subtle differences in brain function and connectivity in adolescents following recovery from mTBI. Newsome et al. identified alterations in functional connectivity following mTBI in youth athletes cleared to return to play (on average, 1-month postinjury) compared with a control group of athletes with orthopedic injuries.^18^ Other studies have found persisting alterations in functional connectivity anywhere from 3 months up to 3 years post-mTBI.^19–22^ While the findings of these studies have been mixed, one network that has been consistently studied has been the default mode network. The default mode network is a collection of brain regions involved in introspection and tends to be more active in the absence of attention-demanding, goal-directed action.^23,24^ Most of the relevant literature is cross-sectional, and the existing longitudinal studies of pediatric mTBI focus on the period immediately following injury.^19^ Further, few have attempted to link persistent subtle motor deficits with lingering alterations in communication between brain regions.

Studies that have attempted to examine persistent motor deficits at the levels of both brain and behavior have all focused on a combination of three networks: the default mode, somatomotor, and dorsal attention. In contrast to the default mode network, somatomotor and dorsal attention regions are more active during goal-directed action. The somatomotor network consists of somatosensory and motor regions of the cortex activated during body movements.^25^ The dorsal attention network consists of regions activated during spatially directed and spatial attention tasks.^25^ Cantarero et al. used transcranial magnetic stimulation of the primary motor cortex to demonstrate impaired behavioral and neurophysiological measures of motor learning in a group of young adults more than 1 year after injury.^26^ In addition, two studies focusing on youth within 3 months of injury identified associations between PANESS performance and functional connectivity of the somatomotor network with the dorsal attention and default mode networks. Risen et al. found that, in youth recovered from mild to moderate TBI, increased somatomotor-dorsal attention connectivity was associated with better motor performance and increased somatomotor-default mode connectivity was associated with poorer motor performance on the PANESS.^27^ A more recent, larger study by Crasta et al. reported similar associations between somatomotor-default mode connectivity and motor performance in youth clinically recovered from mTBI.^28^ Discrepant to Risen et al.’s findings,^27^ Crasta et al. reported increased somatomotor-dorsal attention connectivity associated with worse motor performance on the PANESS across all participants with a stronger association in youth recovered from mTBI than in controls.^28^ Taken together these studies suggest that changes in communication among these networks may be associated with persisting motor deficits. However, these cross-sectional papers study a mixture of acute and chronic phases of mTBI in youth.

There is currently a lack of understanding about serial changes in functional connectivity and subtle motor performance in the period following clinical recovery. We aimed to address this gap by characterizing the longitudinal trajectory of persisting brain and behavioral signs following clinical recovery. Expanding upon prior research, we focused on longitudinal changes in somatomotor-dorsal attention and somatomotor-default mode connectivity. For any group differences in functional connectivity, we continued to explore how these changes were associated with subtle motor performance, as measured by the PANESS.

## Methods

### Procedure

The Johns Hopkins Medicine Institutional Review Board approved this research. Written informed consent was obtained from parents or guardians, and assent was obtained from all child participants. Participants aged 10–17 years were recruited as part of an ongoing longitudinal study of brain recovery after mTBI in youth. Eligible participants were classified into one of two groups: (1) an mTBI group with a recent history of mTBI or concussion, who were enrolled after clinical recovery defined by symptom resolution, subjective return to baseline, and clearance from a medical team to return to all typical activities or (2) a control group of never-concussed, neurotypical participants. For mTBI participants, clinical recovery needed to occur within 3 months of injury (representing typical recovery after youth concussion^29^) and the initial study visit was scheduled within 60 days after clinical recovery from the mTBI.

Participants were recruited for a larger study looking at longitudinal brain–behavior relationships in subtle motor performance after clinical recovery from mTBI. Participants were excluded from the larger study if they had developmental, mental, or learning disorders (e.g., attention-deficit/hyperactivity disorder, anxiety, depression). Participants from the larger study who completed behavioral testing and brain imaging that included task-based and resting-state functional magnetic resonance imaging (fMRI) scans at an initial visit and at a 3-month follow-up visit were included in this study.

### Measures: Behavioral testing for subtle motor signs

We used the Revised PANESS^11^ to assess subtle motor performance at the initial visit and the 3-month follow-up visit. The PANESS examines subtle signs of motor impairment during gait, balance, and timed basic movements. For this study, we used scores from the timed repetitive tasks (e.g., bilateral foot tapping, hand patting, and finger tapping) and sequential tasks (e.g., bilateral heel-toe tapping, hand pronate/supinate, and finger succession). The total time in seconds taken to complete both repetitive and sequential tasks is summed to compute a single PANESS total raw time score that captures speed and accuracy deficits.^30^ We focused on the PANESS total raw time measure based on prior research that has recommended this measure and found it to be sensitive in detecting longitudinal changes in subtle motor performance.^30–32^ Higher PANESS total raw time indicates that participants took longer to complete the repetitive and sequential tasks, reflecting poorer subtle motor performance. A trained neuropsychologist, psychology associate, or masters-level research assistant administered and scored the PANESS at both visits.

### Measures: Sport participation and sociodemographic questionnaires

We asked parents of participants to complete a survey describing their child’s lifetime participation in sports given prior research showing relationships between advanced sport participation and both PANESS performance^12^ and functional connectivity.^33,34^

For the current study, we used advanced sport participation as an *a priori* covariate. Advanced sport participation was defined as participating in at least one school sport (requiring try-outs and typically involving daily practice during the sport season and regular local competitions) or travel sport (typically involving at least weekly practices during the sport season or year-round and competing at the regional or national level). Nonadvanced sport participation was defined as a recreational level of sport participation or no participation in any sports.^12^

Parents also filled out basic sociodemographic information including a maternal education questionnaire indicating the highest level of education completed by the child’s mother at the initial visit. Recent research has reported associations between maternal education and inter- and intranetwork functional connectivity, including the dorsal attention network.^35,36^ Given these findings, we aimed to test for group differences in maternal education in addition to age, sex, race, and sport participation.

### Imaging procedure

Participants completed a task-based fMRI scan, followed by a resting-state scan at their initial and 3-month follow-up visits. Prior to the initial visit scan, all participants underwent a brief training session in a mock scanner. The aim of the training session was to acclimatize them to the MRI environment and allow them to practice the finger-tapping sequence required for the task-based fMRI scan while remaining still. For the finger-tapping sequence of the task-based scan, participants tapped the tips of their index, middle, ring, and pinky fingers, in that order, to the tip of their thumb. Participants were instructed to tap in this sequence repeatedly as accurately and as quickly as they could until they were prompted to stop, marking the end of one tapping block. They completed four blocks of tapping with each hand, with short breaks in between during which they did not tap at all. A screen prompted participants on which hand they should begin tapping with (left or right) and when to take a break. For the resting-state scan, participants were instructed to relax and focus on a centrally located crosshair mark on the screen.

### Scan acquisition and processing

Imaging was performed on a 3T Philips Achieva MRI Scanner (Philips Healthcare, Best, Netherlands) with a 32-channel head coil. Task-based and resting-state fMRI scans both used a single shot, partially parallel, gradient-recalled echo planar imaging (EPI) sequence (Echo Time (TE) = 30 ms, Repetition Time (TR) = 2.5 s, flip angle = 75°, Field of View (FOV) = 256 × 256 mm, a matrix size of 84 × 84, first four TRs were discarded for magnetization stabilization). Within each volume, an ascending slice order was used to collect 47 3-mm axial slices with no slice gap. For task runs, 216 volumes were acquired; for resting-state runs, 156 (duration: 9 and 6.5 min, respectively).

Consistent with current best practices, we aggregated task-based and resting-state data to optimize the reliability of our functional connectivity analysis.^37^ The reliability of functional connectivity is known to be impacted by scan length, suggesting the need for more data per participant to improve uncertainty.^38^ Task effects on functional connectivity across individuals are minimal,^39^ and the improvement in functional connectivity reliability by concatenating task-based and resting-state data outweighs the cost of additional variability introduced by the task.^40^

Each participant’s task-based and resting-state functional scans were subject to a quality control (QC) procedure. QC guidelines were as follows: (1) The task-based and resting-state scans each needed to have at least 2 consecutive minutes or 48 dynamics of low motion data. Low motion data were defined as movement <3 mm in three translational and three rotational directions and (2) the total scan time (task-based plus resting-state) needed to be at least 5 min or 120 dynamics of low motion data after removal of segments with movement >3 mm. Framewise displacement was also calculated from the rigid body realignment parameters^41^ as a summary metric of participant head motion.

Both tasked-based and resting-state fMRI data were preprocessed using Statistical Parametric Mapping (SPM12) and custom scripts (https://github.com/KKI-CNIR/CNIR-fmri_preproc_toolbox) written in MATLAB version 2018a (The Mathworks Inc, Nak tick, MA). Scans were slice-time corrected using the slice acquired at the middle of the TR as reference. Motion realignment parameters were estimated by treating the brain as a rigid body and aligning all volumes to the first volume in the session and then aligning to the mean volume in the second step. fMRI data were directly normalized to the EPI Montreal Neurological Institute template with 2-mm isotropic resolution using the middle volume.^42^ Temporal linear trends were subsequently removed on a voxel-wise basis. The aCompCor50 method was used for estimating nuisance signals from white matter and ventricles.^43^ Nuisance signals along with linearly detrended versions of the six motion parameters (X, Y, Z, Pitch, Roll, Yaw) and their first derivatives (estimated through backward differences) were regressed from each voxel. Scans were then spatially smoothed using a 6-mm full width at half maximum Gaussian kernel. Last, temporal bandpass filtering was applied to constrain signals between 0.01 and 0.1 Hz.

**FIG. 1.**
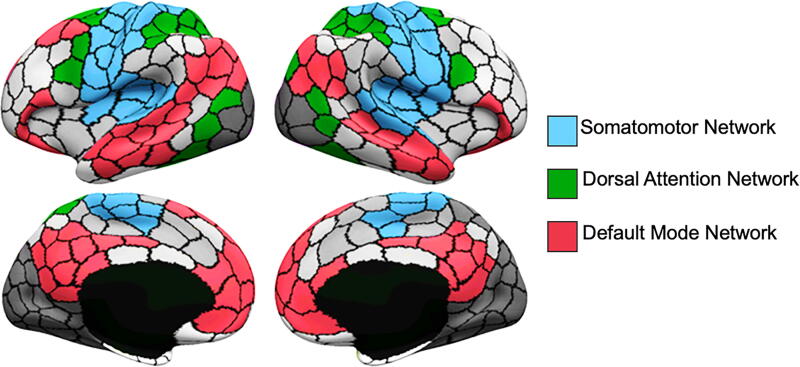
Visualization of the somatomotor, dorsal attention, and default mode networks based on a publicly available hierarchical atlas.^25,44^

An atlas-based correlation analysis was used to probe functional connectivity between somatomotor and dorsal attention brain regions and between somatomotor and default mode brain regions. Somatomotor (blue regions in Fig. 1), dorsal attention (green regions in Fig. 1), and default mode (red regions in Fig. 1) functional networks were defined using a publicly available, hierarchical atlas.^25,44,45^ Residual mean blood oxygenation level-dependent (BOLD) time series were extracted from these three functional networks separately for the resting-state and task-based scans. Functional connectivity was estimated by computing Pearson’s correlation coefficient (*r*) between the mean BOLD time series for the pairs of networks. Correlation coefficients were subsequently *r*-to-*z* transformed. Fisher-*Z* scores from the task-based and resting-state scans of each participant were averaged to obtain an aggregated somatomotor-dorsal attention and somatomotor-default mode functional connectivity estimate.^40^

### Confidence intervals for visit connectivity estimates

We aimed to determine the extent to which observed longitudinal changes in functional connectivity point estimates reflected genuine neurobiological alterations versus sampling error. To address this question, we adopted a bootstrapping resampling technique to calculate average confidence intervals across participants for connectivity estimates at initial and 3-month follow-up scans.

For each task-based or resting-state fMRI scan, we resampled average timeseries from the somatomotor, dorsal attention, and default mode networks with replacement 1000 times. The statistical parameter of interest, a Fisher-*Z* transformed correlation coefficient, was calculated from each of the 1000 samples for somatomotor-dorsal attention and somatomotor-default mode. This process was repeated for each participant’s task-based and resting-state scans at initial and follow-up visits. We averaged task-based and resting-state functional connectivity estimates for each resampling to obtain a distribution of 1000 aggregated estimates that were used to calculate standard error and 95% confidence intervals for each participant at each visit (e.g., initial and 3-month follow-up). Kudela et al. established the validity of a similar block bootstrapping procedure with timeseries data in generating estimates of time-varying connectivity and their associated uncertainty levels.^46^ R Version 4.2.0^47^ (R Core Team, 2022) was used to conduct these analyses.

### Comparing categorical trajectories in functional connectivity and PANESS performance

Each participant was assigned a trajectory to reflect the direction of change in somatomotor-dorsal attention and somatomotor-default mode connectivity from initial to 3-month scans. Participants who had more positive connectivity estimates at their 3-month scan were assigned an “Increase” trajectory, and those with less positive connectivity estimates at 3 months were assigned a “Decrease” trajectory. We calculated the distribution of these trajectories in mTBI and control participants to investigate if groups were more likely to show increases or decreases over time. Group differences in the distribution of somatomotor-dorsal attention and somatomotor-default mode connectivity trajectories were assessed using Fisher’s exact test.

For pairs of networks that we found group differences in, we further explored whether categorical changes in functional connectivity were related to categorical changes in subtle motor performance. We assigned each participant a category that described how their functional connectivity and performance on the PANESS changed over time. The categories reflected if connectivity estimates increased or decreased and if the PANESS total raw time was faster or slower at the 3-month follow-up than at the initial visit. We assigned participants to one of the following four categories: (1) decrease in connectivity and slower PANESS, (2) increase in connectivity and slower PANESS, (3) decrease in connectivity and faster PANESS, and (4) increase in connectivity and faster PANESS. Group differences in the distribution of categories reflecting change in somatomotor-dorsal attention connectivity and PANESS total raw time were assessed using Fisher’s exact tests. These analyses were performed using the Statistical Package for Social Sciences, Version 28.0.

### Comparing dimensional trajectories in Somatomotor-Dorsal attention connectivity and PANESS performance

To further investigate these brain–behavior relationships, we also considered dimensional measures of changes in functional connectivity and subtle motor performance. Delta scores reflecting quantitative change in somatomotor-dorsal attention connectivity and in PANESS total raw time were calculated. Δsomatomotor-dorsal attention connectivity and ΔPANESS total raw time were defined, respectively, as follows:

Δ functional connectivity = functional connectivity at 3 months-functional connectivity at Initial

Δ PANESS total raw time = PANESS total raw time at 3 months-PANESS total raw time at Initial

Based on these formulas, a more positive Δfunctional connectivity score indicates an increase in connectivity at 3 months compared with the initial visit. A more positive ΔPANESS total raw time score indicates that the participant took longer to complete the repetitive and sequential domains of the PANESS and therefore had a poorer PANESS score at 3 months compared with initial. We used a multivariate linear regression model to investigate dimensional change in PANESS performance as a function of dimensional change in functional connectivity. We included an interaction term to evaluate group differences in this relationship, as well as terms for the following potential confounders: sex, age, and sport participation. These analyses were performed using R Version 4.2.0 (R Core Team, 2022). Data were inspected for outliers above 3.29 standard deviations, assessed for normality, and tested for the appropriate assumptions per statistical analysis.

## Results

Sociodemographics, injury, and scan quality profiles of sample participants are detailed in Table 1 and discussed below.

**Table 1.** Sociodemographics, Scan Quality, and Injury Profiles of Participants by Group

	mTBI (*N* = 12)		Controls (*N* = 29)			
	Mean	SD	Mean	SD	*U*	*p*
*Participant demographics*						
Age (years)						
At initial visit	13.83	2.79	13.05	2.36	209.5^a^	0.314
At 3-month visit	14.09	2.77	13.32	2.38	208.0^a^	0.342

	*N*	%	*N*	%	X^2^	*p*
Sex						
Male	7	58.3%	15	51.7%	0.150^b^	0.485
Female	5	41.7%	14	48.3%		
Race						
White	11	91.7%	22	75.9%	2.164^b^	0.673
Black or African American	1	8.3%	2	6.9%		
Asian	0	0%	1	3.4%		
More than one race	0	0%	4	13.8%		
Maternal Ed.						
Less than high school	0	0%	2	6.9%	2.626^b^	0.811
Some college; no degree	2	16.7%	0	0%		
Associate degree	1	8.3%	1	3.4%		
Bachelor’s degree	4	33.3%	8	27.6%		
Master’s degree	2	16.7%	9	31.0%		
Doctoral degree	3	25.0%	6	20.7%		
Advanced Sport Participation						
Advanced sport participator	12	100%	15	51.7%	8.797^a^	0.002^*^
Not an advanced sport participator	0	0%	14	48.2%		

	Mean	SD	Mean	SD	*U*	*p*
*Scan characteristics*						
Framewise displacement						
At initial visit	0.3280	0.3125	0.3516	0.2427	138.0^a^	0.314
At 3-month visit	0.3390	0.2755	0.3422	0.2429	173.0^a^	0.989
Task-based scan time (min)						
At initial visit	7.965	1.885	7.892	2.378	174.0^a^	1.00
At 3-month visit	8.608	1.360	8.614	1.468	171.5^a^	0.944
Resting-state scan time (min)						
At initial visit	5.882	1.476	6.269	0.7534	161.0^a^	0.724
At 3-month visit	6.240	0.9007	6.361	0.5879	170.5^a^	0.944
Total scan time (min)						
At initial visit	13.85	3.214	14.16	2.845	171.0^a^	0.944
At 3-month visit	14.848	2.260	14.97	1.649	172.0^a^	0.966

	Mean	SD	Mean	SD	*t*	*p*
*PANESS performance*						
PANESS total raw time (s)						
At initial visit	64.83	11.36	67.52	12.27	−0.548^c^	0.587
At 3-month visit	63.18	5.63	60.59	9.04	0.917^c^	0.365
*Injury characteristics*						
Days since mTBI						
At initial visit	76.33	35.83				
At 3-month visit	174.0	33.78				
Days since clearance						
At initial visit	35.83	15.33				
At 3-month visit	133.0	19.44				

For continuous variables, mean and standard deviation (SD) are indicated; Mann–Whitney *U* tests were used to assess group differences. For binary and categorical variables, frequencies and percentages are summarized, and differences between groups were assessed using the chi-square test. Groups differed significantly based on sport participation. Scan quality was similar across groups and both initial and 3-month follow-up visits.

^a^ Independent sample Mann–Whitney U test.

^b^ Chi-square test.

^c^ Independent sample *t*-test.

^*^ Significant at the 0.05 level.

mTBI, mild traumatic brain injury; PANESS, Physical and Neurological Examination of Subtle Signs.

### Participant sociodemographics and injury characteristics

Forty-two participants (13 mTBI) completed the task-based and resting-state scans at an initial and 3-month visit, and all scans met QC criteria. However, *n* = 1 (mTBI) was excluded because of a scheduling conflict resulting in the latter visit being conducted 8 months after the initial visit, and *n* = 1 (control) participant was excluded only from analyses with PANESS scores because their ΔPANESS total raw time was an outlier (*Z* = −3.88). The final sample included 12 youth in the mTBI group (*M*_age at Initial Visit_ = 13.83 years, 58% male) and 29 never-concussed controls (*M*_age at Initial Visit_ = 13.05 years, 51% male). Mean number of days between the initial and 3-month follow-up visit was 97.37 (standard deviation [SD] = 12.05 days, range = 79–126 days).

No significant group differences were found based on age, sex, race, or maternal education. Groups differed significantly based on participation in advanced sports (*p* = 0.002). All 12 mTBI participants were advanced sport participators, while only 52% of the controls were advanced sport participators. For the mTBI group, the mean number of days between the mTBI and the initial visit was 76.33 (SD = 35.83, range = 24–199 days), and the mean number of days between clinical recovery and the initial visit was 35.83 (SD = 15.33, range = 3–59 days).

### Scan quality characteristics

No participants were excluded due to scan quality at either visit, although some fMRI frames were removed if movement >3 mm was recorded. At each visit, mean framewise displacement was not significantly different between groups (details in Table 1). Mean framewise displacement also did not differ significantly at the 3-month follow-up scan compared with the initial scan for the mTBI (*p* = 0.624) or the control (*p* = 0.608) group. Within a visit, groups did not differ, on average, in total usable scan time (task-based + resting state), usable task-based time, or usable resting-state time (details in Table 1). Within each group, total usable scan time, usable task-based time, and usable resting-state time did not differ on average between visits (*p* > 0.08 for all comparisons).

### Group differences in categorical trajectories of somatomotor-dorsal attention connectivity and PANESS performance

**FIG. 2.**
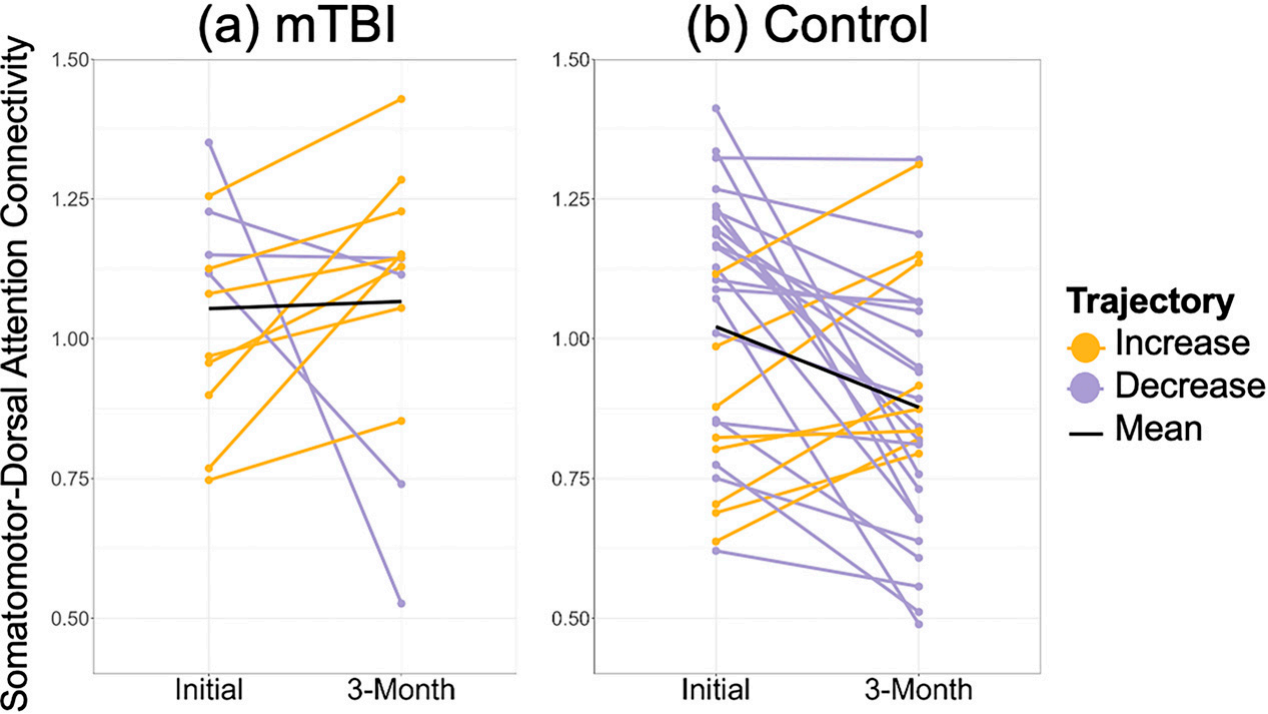
Group differences in the distribution of functional connectivity categorical trajectories from initial to 3 months. Control participants (right panel) were more likely to show a decrease in somatomotor-dorsal attention functional connectivity between visits compared with participants with a history of mild traumatic brain injury (mTBI; left panel). Lilac lines represent participants who showed decreased functional connectivity between visits; gold lines represent participants who showed increase functional connectivity between visits; black lines represent the mean trajectory for each group.

Data from all participants were included in this analysis (*n* = 41, 12 mTBI, and 29 control). Functional connectivity between the somatomotor and dorsal attention networks was positively correlated for all participants at both visits. Figure 2 illustrates individual trajectories paneled by group.

The distribution of somatomotor-dorsal attention categorical trajectories from initial to 3-month differed significantly between participants recently recovered from mTBI and controls (*p* = 0.04). Control participants were more likely to show a decrease (lilac lines in Fig. 2) in somatomotor-dorsal attention connectivity from the initial to 3-month follow-up visit. In contrast, 66.7% of participants recovered from mTBI showed an increase in connectivity between visits (gold lines in Fig. 2). Table 2 summarizes these group differences. The distribution of somatomotor-default mode categorical trajectories from initial to 3 months did not differ significantly between participants recently recovered from mTBI and controls (*p* = 0.26). All subsequent analyses focused on changes in somatomotor-dorsal attention connectivity.

**Table 2.** Categorical Classification of Somatomotor-Dorsal Attention Connectivity and PANESS Performance Trajectories

	mTBI (*n* = 12)	Controls (*n* = 29)	*p*
Somatomotor-dorsal attention connectivity			
Increase in connectivity	8 (66.7%)	8 (27.6%)	0.034^a^^,^^*^
Decrease in connectivity	4 (33.3%)	21 (72.4%)	

	mTBI (*n* = 12)	Controls (*n* = 28)	*p*
Somatomotor-dorsal attention connectivity and PANESS total raw time			
Decrease in connectivity and slower PANESS	1 (8.33%)	5 (17.9%)	0.026^a^^,^^*^
Increase in connectivity and slower PANESS	6 (50.0%)	2 (7.14%)	
Decrease in connectivity and faster PANESS	3 (25.0%)	15 (53.6%)	
Increase in connectivity and faster PANESS	2 (16.67%)	6 (21.4%)	

Frequencies and percentages are summarized for each group. PANESS performance was measured by the PANESS total raw time, which is a sum of total time on repetitive tasks and total time on sequential tasks in seconds. On average, control participants were more likely to show a decrease in somatomotor-dorsal attention functional connectivity between visits compared with the mTBI group.

^a^ Fisher’s exact test.

^*^ Significant at the 0.05 level.

mTBI, mild traumatic brain injury; PANESS, Physical and Neurological Examination of Subtle Signs.

**FIG. 3.**
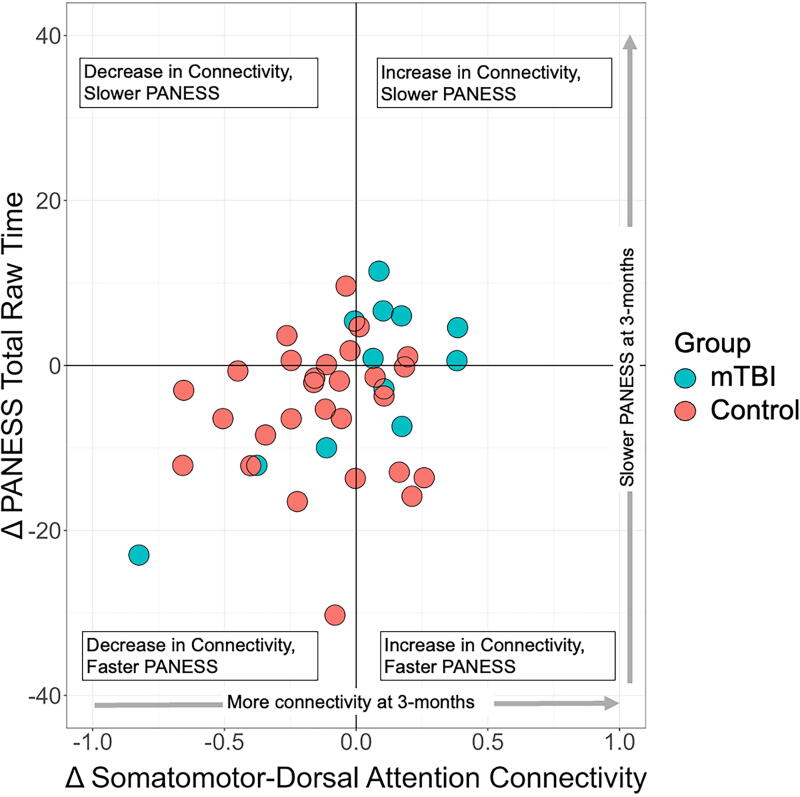
Visual representation of the distribution of categorical change in somatomotor-dorsal attention connectivity and change in PANESS total raw time. Majority mTBI participants (blue dots) demonstrated increased connectivity and slower PANESS performance at 3 months compared with their initial visit. Majority control participants (red dots) showed decreased connectivity and faster PANESS performance at 3 months compared with their initial visit. mTBI, mild traumatic brain injury; PANESS, Physical and Neurological Examination of Subtle Signs.

We further investigated whether the categorical change in somatomotor-dorsal attention functional connectivity was related to the categorical change in PANESS performance. Figure 3 illustrates the distribution of participants in the four possible categories of functional connectivity-PANESS change while Table 2 summarizes frequencies, percentages, and group differences. Data from *n* = 40 (12 mTBI and 28 Controls) participants were included in this analysis. The distribution of categories differed significantly between groups (*p* = 0.02). Of the control participants, 53.5% showed decreases in connectivity with a faster PANESS score at 3 months. In contrast, 50.0% of mTBI participants showed an increase in connectivity with a slower PANESS score at 3 months (see Table 2).

### Group difference in dimensional trajectories for somatomotor-dorsal attention connectivity and PANESS performance

Data from *n* = 40 (12 mTBI and 28 Controls) participants’ scans and PANESS performance were included in this analysis. Mean somatomotor-dorsal attention connectivity and 95% confidence intervals for connectivity estimates were included in this analysis to gauge the extent to which change in connectivity was a product of genuine neural functioning as opposed to sampling error. Mean somatomotor-dorsal attention connectivity for the whole sample at the initial visit was 1.03 (SD = 0.217) with an average confidence interval of 0.0080 and at the 3-month follow-up was 0.451 (SD = 0.091) with an average confidence interval of 0.0076.

**FIG. 4.**
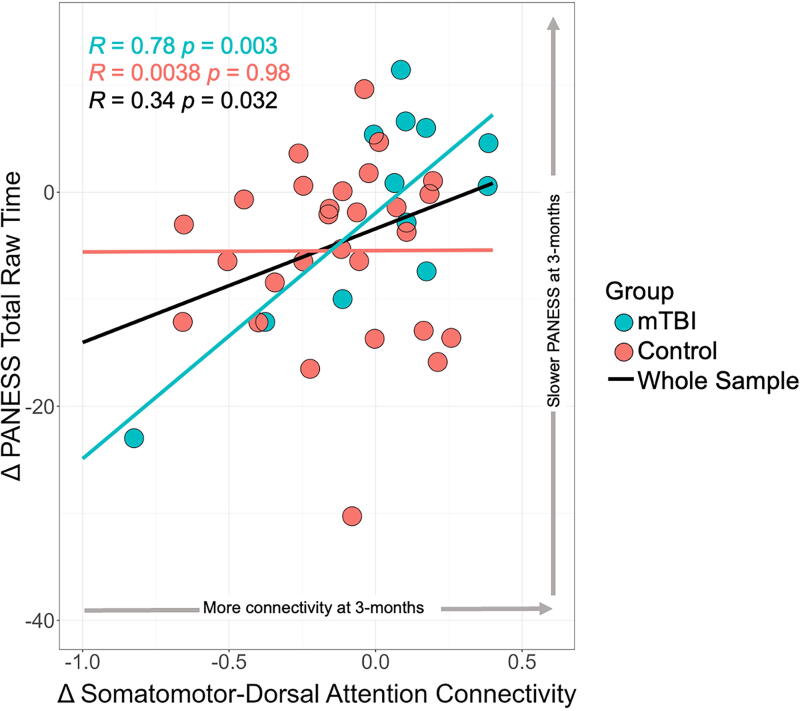
Impact of group on the relationship between dimensional change in subtle motor performance and dimensional change in somatomotor-dorsal attention connectivity. There was a significant relationship between Δsomatomotor-dorsal attention connectivity (*x*-axis) and ΔPANESS total raw time (*y*-axis) for the whole sample (black line) such that an increase in somatomotor-dorsal attention connectivity was associated with slower PANESS total raw time at 3 months compared with the initial visit. Correlations by group indicated that this relationship between Δsomatomotor-dorsal attention connectivity and ΔPANESS total raw time was stronger in the mTBI group (blue line) than in the control group (red line). mTBI, mild traumatic brain injury; PANESS, Physical and Neurological Examination of Subtle Signs.

Our overall linear regression model of ΔPANESS total raw time was significant (*F*[6, 33] = 3.51, *p* < 0.01), with an adjusted *R*^2^ value of 0.278 (see Table 3). Further, the effect of the interaction between group and Δsomatomotor-dorsal attention connectivity on the ΔPANESS total raw time was significant, which is apparent in Figure 4. Participants in the mTBI group who showed a larger increase in connectivity at the 3-month follow-up relative to the initial visit were more likely to perform the PANESS timed tasks more slowly at the 3-month follow-up relative to the initial visit. Relative to controls, a 0.1 increase in Δsomatomotor-dorsal attention functional connectivity resulted in a 1.984 s increase in ΔPANESS total raw time (β = 0.941, *t*[33] = 2.18, *p* < 0.05). There were no significant main effects for Δsomatomotor-dorsal attention connectivity (*p* = 0.12), group (*p* = 0.43), sex (*p* = 0.67), or sport participation (*p* = 0.79) individually. There was, however, a significant main effect of age at the initial visit on ΔPANESS total raw time. A 1-year increase in age resulted in a 1.29 sec change in ΔPANESS total raw time (β = 0.369, *t*[33] = 2.57, *p* < 0.05).

**Table 3.** Dimensional Change in PANESS Performance as a Function of Dimensional Change in Somatomotor-Dorsal Attention Functional Connectivity and Group Membership (History of mTBI or Never-Injured Control)

	*B*	β	Std. error	*t*	*p*
Dependent variable: ΔPANESS total raw time					
Constant	−25.89		7.370	−3.512	0.001^**^
Δsomatomotor-dorsal attention connectivity	−21.27	−0.680	13.57	−1.567	0.126
Group	2.375	0.125	2.981	0.797	0.431
Sex	1.064	0.061	2.471	0.430	0.670
Age	1.293	0.369	0.503	2.571	0.015^*^
Sport participation	0.755	0.041	2.887	0.262	0.795
Δsomatomotor-dorsal attention connectivity^*^ group interaction	19.84	0.941	9.070	2.188	0.036^*^
* R*-squared	0.390				
Adjusted *R*-squared	0.279				
Residual standard error	7.47 on 33 degrees of freedom				
* F*-statistic	3.512 on 6 and 33 degrees of freedom				
* p*	0.0085				

PANESS performance was measures by the PANESS total raw time, which is a sum of the total time on repetitive tasks and total time on sequential tasks in seconds. Linear regression was used to model this relationship. mTBI group was the reference group.

^*^ Significant at the 0.05 level.

^**^ Significant at the 0.01 level.

mTBI, mild traumatic brain injury; PANESS, Physical and Neurological Examination of Subtle Signs.

## Discussion

To better understand why adolescents are more prone to longer recovery times and repeat injuries after mTBI, we examined longitudinal changes in functional connectivity and their association with subtle motor performance measured by the PANESS in adolescents clinically recovered from mTBI and never-injured controls. We performed categorical and dimensional analyses, and in both cases, we observed persistent differences between adolescents recovered from mTBI and never-injured controls. Categorically, participants with a history of mTBI were less likely than controls to demonstrate decreases in somatomotor-dorsal attention connectivity over time. Moreover, we found that categorical changes in functional connectivity over 3 months following clinical recovery were related to categorical changes in PANESS performance during this time. From the first to the second visit, participants with a history of mTBI were more likely to exhibit increased somatomotor-dorsal attention connectivity and poorer subtle motor performance compared with controls. Dimensionally, participants post-mTBI who showed a larger increase in somatomotor-dorsal attention connectivity at the 3-month follow-up relative to the initial visit were more likely to perform the PANESS timed tasks more slowly at the 3-month follow-up relative to the initial visit.

While we did not observe differences in functional connectivity or motor performance when comparing groups cross-sectionally, the observed longitudinal group differences in functional connectivity and their association with motor performance could represent a potential vulnerability to future injury. Although, on average, the groups differed in their functional connectivity trajectories, neither the control nor the mTBI group was entirely homogeneous in their patterns of change. Participants in both groups demonstrated increases and decreases in somatomotor-dorsal attention functional connectivity over time, and participants in both groups may or may not sustain future injury. Incorporating analyses of individual-level trajectories may be more informative than solely focusing on group-level findings. It is critical that we continue to follow-up with these participants to see if early increases in somatomotor-dorsal attention functional connectivity are predictive of future injury in both youth who were never injured and those clinically recovered from mTBI. In addition, our motor task may not have been challenging enough to elicit behavioral differences at the group level at either visit. Prior research suggests that more complex tasks, particularly those requiring participants to complete cognitive and motor tasks simultaneously, have been sensitive in identifying cross-sectional motor deficits in youth recovering from concussion.^48–50^ Such tasks may be more representative of the complex motor and attention tasks often required for everyday living including within the context of sports.^51,52^

Another benefit to our longitudinal design was repeated exposure to the PANESS and the in-scanner tapping task, potentially enhancing our understanding of learning effects in both groups. The decreases in functional connectivity and improved PANESS performance observed in the never-injured youth could suggest neural efficiency and learning within the context of the motor task. Previous research in neurotypical adults has shown that functional connectivity between somatomotor and nonsomatomotor brain regions decreases with the practice of a motor task and that individuals who exhibit greater functional segregation between somatomotor and nonsomatomotor regions following practice were better able to learn than individuals who maintained a consistent level of functional connectivity between somatomotor and nonsomatomotor brain regions.^53^ In addition, increased functional connectivity in the context of pediatric mTBI has been theorized to reflect either a compensatory mechanism or the recruitment of additional neural resources to meet attentional demands.^54,55^ This increased effort, observed in our findings, could indicate a lack of neural efficiency for youth clinically recovered from mTBI. This theory aligns with previous research by Cantarero et al., who reported impaired motor skill learning and dysfunctional communication between motor regions in collegiate athletes, even exceeding 1 year postinjury.^26^ Given that we found a main effect of age on motor performance, future mTBI research in this developmental age range should examine the influence of age on longitudinal changes in performance, which may reflect motor learning.

Although previous research has not focused on longitudinal change over time, some studies have reported cross-sectional associations between somatomotor-dorsal attention connectivity and PANESS performance. Crasta et al. reported a similar association between increased somatomotor-dorsal attention connectivity and poorer performance on the PANESS in a sample of youth postclinical recovery from mTBI (on average, 80 days postinjury) and never-injured controls.^28^ Notably, this association was found to be stronger in youth who had recovered from mTBI compared with controls. However, in contrast to Crasta et al.,^28^ Risen et al. found that increased somatomotor-dorsal attention connectivity was associated with improved PANESS performance in youth postmild to moderate brain injury (on average, 68 days postinjury).^26^ This implies that severity of injury, time since injury, and time since clinical recovery may play a role in how functional connectivity and subtle motor performance are associated.

While a growing body of research in pediatric mTBI has reported alterations in somatomotor-default mode network connectivity, we did not observe these differences longitudinally. This divergence from existing research may be attributed to the inclusion of task-based fMRI scans in our methodology. Because the default mode network is more active during rest, past research has mainly used resting-state scans to study it. By incorporating task-based scans into our design, we may have increased the sensitivity of our data to detect changes within a network crucial for goal-directed action. There is currently a lack of research in pediatric mTBI populations including task-based fMRI analyses for motor-related activation. However, cognitive task-based fMRI research in this population has found altered activation in the middle frontal gyrus,^56^ which is functionally connected to the dorsal and ventral attention networks.^57^ Further, Greene et al. found that cognitive tasks amplify individual differences in functional connectivity associated with behavioral performance.^58^

Our findings may also deviate from existing literature due to potential issues with the quality of imaging data. Functional connectivity data are notoriously noisy, and the changes we observed between the initial and 3-month follow-up visits were small. However, we took several steps to ensure that these changes were real and reliable. First, we compared several scan quality metrics between visits and between groups. Within each group, total usable scan time, usable task-based time, and usable resting-state time did not differ on average between visits (*p* > 0.08 for all comparisons). Within a visit, groups did not differ on average, in total usable scan time, usable task-based time, or usable resting-state time. Overall, the lack of visit or group differences in these scan quality metrics minimizes concerns that observed differences in functional connectivity are solely due to motion artifacts. We also combined task-based and resting-state scan data when calculating functional connectivity point estimates as this has been shown to improve reliability.^40^ Finally, we used a bootstrapping method to calculate confidence intervals for connectivity estimates and found that uncertainty in functional connectivity point estimates (∼0.008) was smaller than the changes in connectivity observed over time.

While this study provides valuable insights into brain–behavior changes following recovery, further research is needed to address potential limitations. The generalizability of our findings is limited because all our youth in the mTBI group were advanced sport participators and demonstrated typical recovery from mTBI. It is unclear if youth who are less active or have prolonged recovery and ongoing symptoms beyond 6 weeks postinjury would show similar brain–behavior changes. Important follow-up questions include whether these brain–behavior changes persist past 3 months after clinical recovery, relate to reinjury and repeat mTBIs, and, whether they can be normalized with motor/attention training.

While we did not observe differences in functional connectivity or motor performance when comparing groups cross-sectionally, the observed longitudinal group differences in functional connectivity and their association with motor performance could still represent a potential vulnerability to future injury.

## Abbreviations Used

FOV: Field of View
mTBI: mild traumatic brain injury
SD: standard deviation
